# Maternal Abnormal Liver Function in Early Pregnancy and Spontaneous Pregnancy Loss: A Retrospective Cohort Study

**DOI:** 10.2188/jea.JE20240233

**Published:** 2025-05-05

**Authors:** Huibin Yang, Tianyi Tang, Qianlei Qian, Xiaohua Zhang, Yinan Liu, Xiaoyan Zhou, Yanling Zhang, Longmei Jin, Xiaotian Chen

**Affiliations:** 1 Minhang Maternal and Child Health Hospital, Shanghai, China; 2 Nantong Third People’s Hospital, Affiliated Nantong Hospital of Nantong University, Jiangsu, China; 3 Department of Respiratory and Critical Care Medicine, The Fifth Affiliated Hospital of Sun Yat-sen University, Zhuhai, China; 4 Department of Clinical Epidemiology & Clinical Trial Unit, Children’s Hospital of Fudan University, National Children’s Medical Center, Shanghai, China

**Keywords:** liver function, early pregnancy, spontaneous pregnancy loss, liver enzyme levels, cohort study

## Abstract

**Background:**

Spontaneous pregnancy loss (SPL) precedes an increased risk of reduced fertility, while its etiology mechanism remains largely unknown. Liver dysfunction presenting in early pregnancy may represent a pre-existing undiagnosed liver condition affecting fetal development. Here, we investigated whether maternal abnormal liver function in early pregnancy contributed to the incidence of SPL.

**Methods:**

Data on pregnant women were leveraged from the Maternal Health Care Information System in Shanghai City from 2017 to 2021. Liver dysfunction status was defined as having any elevated liver function biomarker levels (LFBs) at the first antenatal visit. SPL cases were defined as fetal death occurring before 28 weeks gestation. Generalized linear models were used to estimate crude and adjusted risk ratios (RRs and aRRs, respectively) and 95% confidence intervals (CIs).

**Results:**

Among 10,175 leveraged pregnant women, 918 (9.0%) SPL cases were recorded. Maternal liver dysfunction in early pregnancy was associated with a 49% increased risk of SPL (RR 1.49; 95% CI, 1.22–1.84). This positive association persisted after adjustment for covariates (aRR 1.55; 95% CI, 1.26–1.92). Higher γ-glutamyl transferase (GGT) and alkaline phosphatase (ALP) levels were also linked with increased risk of SPL in a linear fashion (aRRs per 1 standard deviation increase: 1.13; 95% CI, 1.08–1.17 and 1.13; 95% CI, 1.07–1.20, respectively). Similar magnitudes of associations were observed between normal weight and overweight pregnant women in subgroup analysis.

**Conclusion:**

We provide new evidence that maternal abnormal liver function in early pregnancy, as well as GGT and ALP, predisposes to an increased risk of SPL.

## INTRODUCTION

Spontaneous pregnancy loss (SPL) is a serious pregnancy morbidity that precedes an increased risk of reduced fertility.^1^^,^^2^ The definition of SPL varies between countries and international organizations, leading to differing estimates of its prevalence among studies.^3^ Data from a large nationally representative survey in the United States indicated that approximately 20% of clinically recognized pregnancies ended in SPL, which includes stillbirths and ectopic or tubal pregnancies, throughout the whole gestation period.^4^ In contrast, SPL is defined as fetal death before 28 weeks gestation in China, but there is little reliable evidence on the epidemiology of SPL.^5^^,^^6^ To date, although maternal genetic, structural, infectious, endocrine, and immune factors have been reported as potential causes of SPL, the etiology mechanism remains largely unknown.^7^^,^^8^

Pregnant women with metabolic disorders have poor endometrium quality, and difficulties with successful implantation and continued nourishment of the pregnancy, which may influence fetal development.^9^ As a common but easily neglected metabolic disorder in early pregnancy,^10^ liver dysfunction status may represent a pre-existing undiagnosed liver condition that has been exacerbated by the physiological and metabolic stresses of the pregnancy. Previous studies have reported its association with adverse pregnancy outcomes risk, including gestational diabetes mellitus, cesarean delivery, and preterm birth,^11^^–^^13^ but the evidence on SPL is scarce. Liver function is usually characterized by the liver function biomarker levels (LFBs), including γ-glutamyl transferase (GGT), alanine aminotransferase (ALT), alkaline phosphatase (ALP), and aspartate aminotransferase (AST).^14^ In this study, we conducted a hospital-based cohort study to determine whether maternal abnormal liver function in early pregnancy, as well as GGT, ALT, AST, and ALP levels, was associated with subsequent SPL risk. Our findings are helpful in providing a better understanding of the maternal abnormal liver metabolism in the etiology of SPL.

## METHODS

### Data source and study population

This retrospective population-based cohort study used the electronic health record data linked by unique coded identifiers from the Maternal Health Care Information System (MHCIS) of Minghang District in Shanghai City, China.^15^ The MHCIS containing the whole antenatal care examination information of the pregnant women in the local district was set to monitor and identify high-risk pregnant women and to provide standardized health care services. Data elements include the pregnant women with primary and secondary diagnoses, discharge status, demographic and routine clinical variables. Each pregnant woman had only one medical record in the current study, so there were no repeated entries existed. If a woman had multiple pregnancy records, we leveraged the first pregnancy record for the final analysis during the time framework. Informed consent was obtained from all participants before the data was leveraged. All methods were carried out in accordance with relevant guidelines and regulations of Minhang Maternal and Child Health Hospital, and the study protocol was approved by the Institutional Ethics Committee of the Minhang Maternal and Child Health Hospital (HS-02).

We first leveraged the prenatal and delivery data of pregnant women aged more than 20 years or older from January 1, 2017, to December 31, 2021. We then assembled a cohort of pregnant women who had complete medical records of pregnancy complication outcomes (including SPL) throughout pregnancy. Those who had the following conditions were excluded from the primary analysis: with no LFBs tests at the first antenatal visit; with hepatitis or any other liver disease during pregnancy; and with stillbirth. This study is reported according to the Reporting of Studies Conducted using Observational Routinely collected Data reporting guideline.^16^

### Outcomes

We defined the SPL cases as fetal death occurring before 28 weeks’ gestation according to the medical text records of pregnant women from the MHCIS. Pregnancy loss occurring after the participants’ first clinic visit was recorded by the gynecologists according to the Chinese clinical guidelines.^5^ Artificial abortions due to ectopic pregnancy, molar pregnancy, fetal malformation, chromosomal abnormalities, or any clinically recognized disorders diagnosed according to the Prenatal Diagnosis Technology Management Measures (Ministry of Health, China) and Shanghai Genetic Counseling Technology Service Management Measures, which were excluded in this study. We verified the diagnosis record by a personal telephone interview to ensure that all SPL cases were accurate.

### Exposures and covariates

Our primary exposure was the maternal liver function status at the first antenatal visit. According to the maternal healthcare procedures guideline in Shanghai, LFBs have been treated as routine and important antenatal examinations in all obstetrics and gynecology hospitals. We defined the maternal liver dysfunction (binary variable, yes or no) indicated by the routinely examined GGT, ALT, AST, and ALP levels. As no specific cutoffs of LFBs were available for the pregnant women to indicate liver dysfunction, we adopted the reference intervals of the GGT, ALT, AST, and ALP levels for the general adult population to define abnormal liver dysfunction (eTable 1).^17^^–^^20^

Several known or suspected risk factors for SPL were identified and included as covariates in the association analysis.^21^ These factors comprised both continuous and binary variables. Continuous variables included age, pre-pregnancy body mass index (BMI), and gestational weeks. Binary variables included parity (0 or ≥1), self-reported diabetes before pregnancy (yes or no), and history of abortion (yes or no). Maternal pre-pregnancy BMI was calculated through self-reported pre-pregnancy body weight and categorized as normal weight (<24 kg/m^2^) or overweight (≥24 kg/m^2^) according to the definition for the Chinese population.^22^ Gestational weeks at the first antenatal visit of each pregnant woman were determined by routine ultrasound examination, on the assumption that at those gestations all fetuses were average for gestational age in size. Parity, diabetes before pregnancy, and history of abortion were leveraged from the medical system.

### Statistical analyses

Continuous variables were reported as mean (standard deviation [SD]) when normally distributed and median (interquartile range) when not. Categorical variables were summarized as frequencies and percentages. Comparisons between the SPL and non-SPL groups were performed for continuous variables using a two-tailed unpaired Student’s *t* test or Mann–Whitney *U* test depending on normality, and categorical variables were compared with a chi-square test.

Our primary aim was to investigate the associations of maternal abnormal liver function (binary variable) in early pregnancy with the risk of SPL. We first used generalized linear models with binomial family and log link function to estimate crude and adjusted risk ratios (RRs and aRRs, respectively) and 95% confidence intervals (CIs). We also quantified the associations of GGT, ALT, AST, and ALP levels (continuous variables, per SD increment) with SPL risk to explore the dose-response relationship, under the assumption of a linear relationship between exposures and the outcome. We also quantified the associations of categorical LFBs defined using the clinical reference with SPL risk. In the multivariable regression model, we adjusted maternal age, gestational week at enrollment, BMI, diabetes before pregnancy, parity, and history of abortion as covariates. We then presented the Bonferroni adjusted *P* values for the main results on the associations of liver dysfunction and individual LFBs with SPL incidence based on the adjusted models.

In the secondary analyses, we used a three-knot (5^th^, 50^th^, and 95^th^ percentile levels) restricted cubic spline (RCS) regression model fitted in R (*rms* package; R Foundation for Statistical Computing, Vienna, Austria) to assess the potential non-linear relationship of LFBs on SPL risk.^23^ As obesity is a common risk factor for both fatty liver and SPL,^24^^,^^25^ we also explored whether the association of liver dysfunction and individual LFBs with SPL was independent of being overweight, we analyzed the above associations by further stratifying BMI <24 kg/m^2^ and ≥24 kg/m^2^. As there were 709 (6.5%) pregnant women with missing LFBs records after the first antenatal visit, we conducted sensitivity analyses to test the robustness of the main results by using multiple imputations with chained equations, assuming that the data were missing at random. Given pregnant women with advanced age and a history of SPL may be associated with SPL risk,^3^ to ensure our main results were free from the potential bias from these two confounders, we further repeated two primary association analyses restricted to subgroups at low risk of SPL, including pregnant women under 35 years and without abortion history, respectively. Besides, since the current study period included the coronavirus disease 2019 (COVID-19) pandemic period, the impact of COVID-19 on maternal liver function and SPL should have also been considered. We conducted a further subgroup analysis by restricting to the pre-COVID-19 period (the time of LFBs measurement before March 11, 2020).^26^ Statistical significance was set at 2-sided *P* < 0.05. Statistical analyses were performed using Stata version 16.0 (Stata Corp LLC, College Station, TX, USA) and R package (version 3.6.1).

## RESULTS

### Baseline characteristics of the participants

We initially extracted data on 11,045 pregnant women with complete medical records of SPL data, and 10,884 pregnant women were included for analysis after excluding 161 subjects with hepatitis or with any other liver disease, 12 stillbirths, and 123 artificial abortions (eFigure 1). We conducted the primary analysis among 10,175 pregnant women with complete LFBs and SPL records. We summarized the characteristics of the 10,175 eligible pregnant women and the 709 with missing LFBs data (eTable 2), which were in general similar.

The mean age of the pregnant women was 29.8 (SD, 4.2) years, and the average gestation was 13.5 (SD, 2.9) weeks at the first antenatal visit (Table 1). Of the participants, 14.8% were overweight, 22.4% reported a history of abortion, and more than half (56.2%) were primipara, while a small proportion (0.1%) had diabetes before pregnancy. The skewed distributions of maternal LFBs are presented in eFigure 2. Elevated GGT, ALT, AST, and ALP levels were found in 2.8%, 7.5%, 3.3%, and 0.6% of pregnant women, respectively. Overall, 10.0% of pregnant women had abnormal liver function in early pregnancy.

**Table 1.** Characteristics of the study population

Characteristics	Total	SPL	Non-SPL
*N*	10,175	918	9,257
Age, mean (SD), year	30.1 (4.2)	30.1 (3.9)	29.7 (4.2)
Pre-pregnancy BMI, mean (SD), kg/m^2^	21.2 (4.0)	21.3 (3.7)	21.2 (3.8)
Overweight, *n* (%)	1,506 (14.8)	104 (15.5)	1,368 (14.8)
Missing, *n* (%)	119 (1.2)	10 (1.1)	109 (1.2)
Gestational week at first antenatal visit, mean (SD), week	13.5 (2.9)	12.6 (1.9)	13.5 (3.0)
Missing, *n* (%)	70 (0.7)	5 (0.5)	65 (0.7)
Parity, *n* (%)			
0	5,715 (56.2)	588 (64.1)	5,127 (55.4)
≥1	4,380 (43.0)	326 (35.5)	4,054 (43.8)
Missing, *n* (%)	80 (0.8)	4 (0.4)	76 (0.8)
History of abortion, *n* (%)	2,283 (22.4)	179 (19.5)	2,104 (22.7)
Missing, *n* (%)	150 (1.5)	15 (1.6)	135 (1.5)
Diabetes before pregnancy, *n* (%)	12 (0.1)	1 (0.1)	11 (0.1)
Missing, *n* (%)	38 (0.4)	4 (0.4)	34 (0.4)
GGT, median (IQR), U/L	12.0 (9.0–16.0)	13.0 (10.0–19.0)	12.0 (9.0–16.0)
Elevated levels, *n* (%)	284 (2.8)	54 (5.9)	230 (2.5)
ALT, median (IQR), U/L	12.0 (9.0–18.0)	12.00 (9.0–19.0)	12.0 (9.0–18.0)
Elevated levels, *n* (%)	765 (7.5)	80 (8.7)	685 (7.4)
AST, median (IQR), U/L	15.0 (13.0–18.0)	15.0 (13.0–19.0)	15.0 (13.0–18.0)
Elevated levels, *n* (%)	337 (3.3)	42 (4.6)	295 (3.2)
ALP, median (IQR), U/L	44.0 (38.0–52.0)	45.0 (39.0–52.0)	44.0 (38.0–52.0)
Elevated levels, *n* (%)	66 (0.6)	3 (0.3)	63 (0.7)
Abnormal liver function, *n* (%)	1,017 (10.0)	121 (13.2)	896 (9.7)

ALP, alkaline phosphatase; ALT, alanine aminotransferase; AST, aspartate aminotransferase; BMI, body mass index (calculated as weight in kilograms divided by height in meters squared); GGT, γ-glutamyl transferase; IQR, inter-quartile range; SD, standard deviation; SPL, spontaneous pregnancy loss.

Continuous variables are presented as mean (SD) or median (interquartile range), and categorical variables are listed as *n* (%).

There are 918 (9.0%) cases of SPL that occurred before 28 weeks’ gestation after the first antenatal visit. Compared with non-SPL participants, pregnant women with SPL were older and had higher levels of GGT but had smaller gestational weeks and a lower proportion of multipara. There was a significantly higher proportion of women with abnormal liver function and elevated AST in the SPL group than in the non-SPL group (13.2% vs 9.7% and 4.6% vs 3.2%, respectively).

### Associations of maternal liver dysfunction and LFBs with SPL

**Figure 1.**
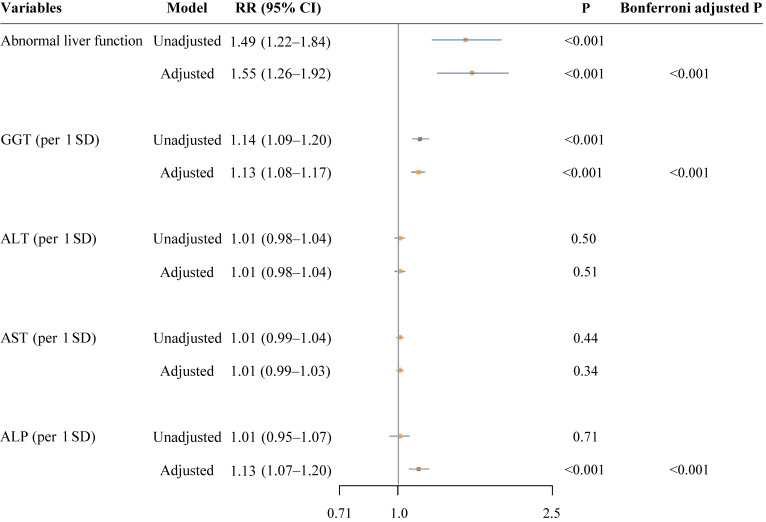
Associations of maternal liver dysfunction and LFBs with SPL risk. ALT, alanine aminotransferase; ALP, alkaline phosphatase; AST, aspartate aminotransferase; CI, confidence interval; GGT, γ-glutamyl transferase; RR, risk ratio; SD, standard deviation; SPL, Spontaneous pregnancy loss.

Pregnant women with abnormal liver function had a 49% increased risk of SPL (RR 1.49; 95% CI, 1.22–1.84; Figure 1). This positive association persisted after adjustment for covariates (aRR 1.55; 95% CI, 1.26–1.92). For respective maternal LFBs, higher GGT and ALP levels were associated with increased risk of SPL (aRRs per 1 SD increase 1.13; 95% CI, 1.08–1.17 for GGT and 1.13; 95% CI, 1.07–1.20 for ALP). These observed positive associations remained significant even after Bonferroni adjustment (all *P* < 0.001). Consistently, the positive associations between categorical GGT and ALP and SPL risk were also observed (aRR 2.34; 95% CI, 1.76–3.11 and 1.72; 95% CI, 1.25–2.38, respectively; eFigure 3). No significant associations of ALT and AST as continuous variables with SPL were observed.

**Figure 2.**
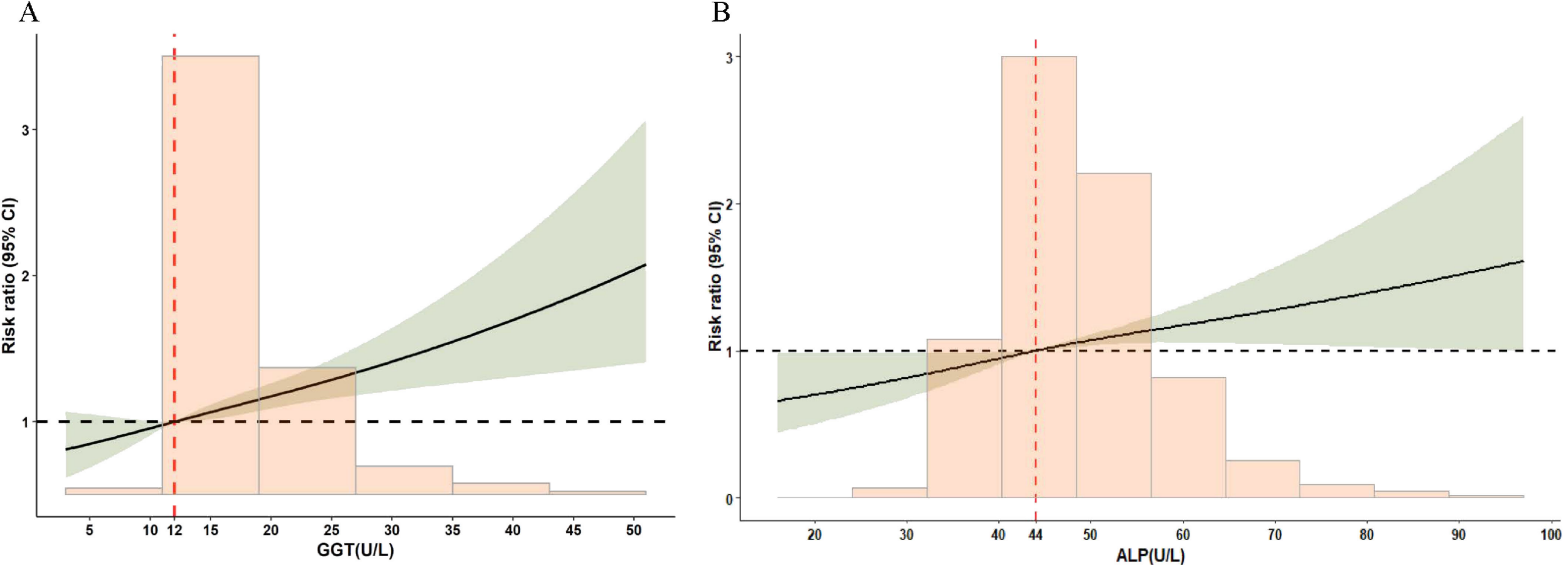
Non-linear association analyses for continuous ALP and GGT levels with SPL risk. The lines indicate estimated risk ratios, and the light green areas represent 95% CI. (**A**) The median level of GGT 12.0 U/L was selected as the reference level. (**B**) Mean of ALP 44.0 U/L was selected as the reference level. The lines indicate estimated risk ratios, and the light green shaded areas represent 95% CIs. ALP, alkaline phosphatase; GGT, γ-glutamyl transferase.

**Figure 3.**
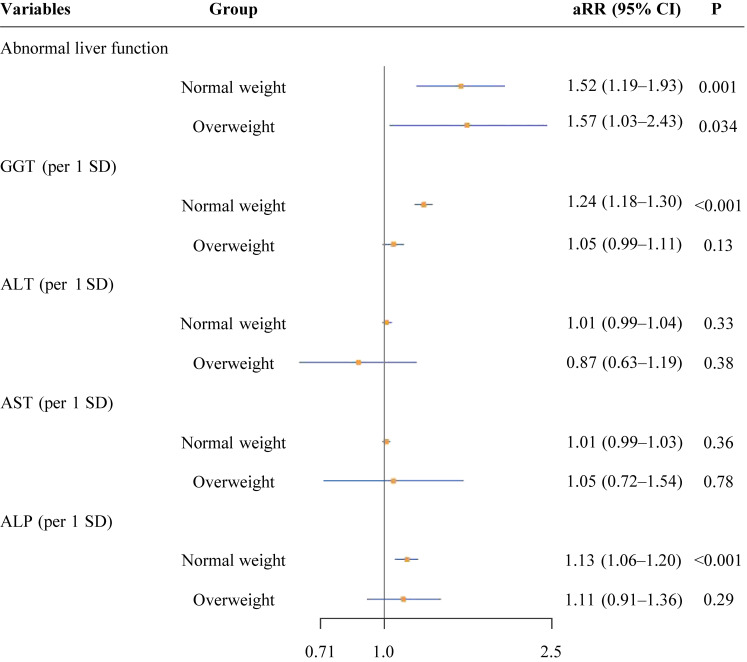
Stratification analysis by overweight status. ALP, alkaline phosphatase; ALT, alanine aminotransferase; aRR, adjusted risk ratio; AST, aspartate aminotransferase; GGT, γ-glutamyl transferase; SD, standard deviation.

In the secondary analyses, RCS regression analyses showed that higher levels of GGT and ALP were associated with increased risk of SPL in a linear fashion (*P* values for non-linearity >0.05; Figure 2). Compared with normal weight pregnant women, the proportion of abnormal liver function was significantly higher in overweight pregnant women (8.9% vs 16.2%; eTable 3). Similarly, the LFBs of overweight pregnant women were significantly higher than those of normal weight subjects (*P* < 0.001). Nevertheless, similar magnitude associations of maternal abnormal liver function were found in both normal weight and overweight pregnant women, respectively (aRRs 1.52; 95% CI, 1.19–1.93 for normal weight pregnant women and 1.57; 95% CI, 1.03–2.43 for overweight pregnant women; Figure 3). In normal weight subjects, per 1 SD increase in GGT and ALP levels were associated with higher SPL risk (aRRs 1.24; 95% CI, 1.18–1.30 for GGT and 1.57; 95% CI, 1.03–2.43 for ALP).

### Sensitivity and subgroup analysis

As shown in eTable 4, when the 709 pregnant women with missing LFBs were included after multiple imputations, the associations between maternal abnormal liver function and SPL risk were similar to the main results (aRR 1.50; 95% CI, 1.26–1.77). We also found that each 1 SD increase in GGT and ALP levels were associated with an 11% and 13% increased risk of SPL, respectively (aRRs 1.11; 95% CI, 1.07–1.16 for GGT and 1.13; 95% CI, 1.07–1.19 for ALP).

In further subgroup analyses restricted to pregnant women under 35 years, with no abortion history, and with LFBs measured before COVID-19 period, the positive associations between abnormal liver function, GGT, and ALP with SPL risk were still significant (eTable 5).

## DISCUSSION

In this population-based cohort study, we provided some evidence that maternal abnormal liver function in early pregnancy is associated with an increased risk of SPL. This association was observed even when GGT and ALP levels were within the normal range in a linear dose-response manner. There was no significant difference in the magnitude of these associations between pregnant women with or without preconception overweight status. Our findings are novel and extend the current knowledge of maternal liver dysfunction on the subsequent pregnancy complications, emphasizing its essential role in SPL incidence.

SPL can be a devastating experience, and there are additional long-term effects on pregnant women, both physical and psychological. Animal studies showed that the female mice with fatty liver caused by a high-fructose diet experienced pregnancy loss and reduced litter size.^27^ Population-based studies found that maternal liver function was associated with a higher risk of gestational diabetes mellitus, cesarean delivery, and preterm birth,^11^^–^^13^ which were consistent with our findings. To our knowledge, this study is the first population cohort study to provide solid evidence linking abnormal liver function in early pregnancy to an increased risk of SPL. Similar positive associations were observed between the continuous GGT, ALP levels, and SPL risk in a linear dose-response manner, as illustrated by our RCS model. Even in the normal range of GGT (≤38 U/L) and ALP (43–115 U/L), both increments in the levels of two biomarkers were associated with SPL risk as shown by the RCS plot. Given the current and previous evidence, we do highlight the need for increased awareness of the risks of SPL in pregnant women due to liver dysfunction in early pregnancy.

Obesity in early pregnancy has been identified as a major contributing factor to SPL.^28^ Meanwhile, abnormal liver function, including hepatobiliary injury and abnormal synthetic function that usually manifests with elevation in LFBs, is known to be a common consequence of obesity.^29^^,^^30^ However, recent studies indicate that abnormal liver function is increasingly observed in non-obese populations as well.^31^^–^^33^ It is of great interest to explore whether obesity could be an important modifier of the association between maternal liver dysfunction and SPL risk. The current study found a similar magnitude of association in the normal weight and overweight pregnant women, suggesting that abnormal maternal liver function associated with SPL may be independent of obesity. However, we remain cautious about this result because lifestyle factors (such as alcohol consumption) and cholestatic conditions were not adjusted in this study. Further basic research or animal studies are warranted to explore the potential mechanisms between maternal liver dysfunction and SPL in non-obese populations.

Our study addresses two critical issues with important clinical and research implications. First, our findings expand on the literature on risk factors for SPL, an adverse pregnancy outcome with poorly understood modifiable determinants. Second, we examine a risk factor that can be modifiable through lifestyle changes and pharmacological interventions. Maternal liver dysfunction in early pregnancy may indicate an undiagnosed pre-existing liver condition exacerbated by the physiological and metabolic stresses of the pregnancy. In clinical practice, GGT serves as a surrogate biomarker for liver steatosis, while both GGT and ALP are indicative of cholestasis.^14^^,^^34^ Unfortunately, imaging examinations or diagnoses of liver steatosis and cholestasis are not available in our study. Several observational studies have reported a significant association of maternal GGT and ALP levels in early pregnancy with the development of gestational diabetes between 24–28 gestation weeks.^13^^,^^35^ Our novel findings contribute new evidence to the relationship between maternal GGT, ALP levels, and SPL risk. These results suggest that maternal liver steatosis or cholestasis may be potential underlying mechanisms for the development of SPL. Typically, physiological changes in LFBs observed during pregnancy are usually mild, transient, and rarely permanent. For instance, ALP increases and may reach two to four times the normal adult upper reference value, while GGT normally remains within the normal range, similar to non-pregnant women.^36^ Based on our findings, an elevation of GGT levels in early pregnancy appears to be a more sensitive indicator of an increased risk of SPL. Therefore, we propose that gynecologists should be vigilant about the severe pregnancy outcomes caused by liver dysfunction that is unrelated to physiological changes in LFBs in early pregnancy, for the latter condition may lead to a severe threat to fetal survival.^37^

The biological mechanism by which liver dysfunction contributes to SPL is unclear. Previous studies have suggested that maternal insulin resistance around conception may contribute to SPL.^38^ Notably, pregnant women with liver dysfunction exhibit a high level of homeostasis model assessment of insulin resistance,^12^ supporting the assumption that insulin resistance during early pregnancy may mediate the association between liver dysfunction and SPL. Besides, adiponectin levels have been observed to be lower in pregnant women with liver dysfunction compared to healthy pregnant women.^12^ Adiponectin plays a crucial role in reducing the embryonic loss rate by improving insulin resistance and inhibiting apoptosis. It achieves this by activating the AMP-activated protein kinase pathway and the PI3K/Akt signaling pathway.^39^ Further studies are warranted to determine the exact mechanism by which liver dysfunction leads to the development of SPL.

We took advantage of a large sample size with comprehensive measurements of LFBs, which enabled us to identify the associations of maternal GGT and ALP levels in early pregnancy with SPL risk. However, several notable limitations exist. First, residual confounding cannot be ruled out, as we did not have data on some covariates, including lifestyle factors (such as alcohol consumption and tobacco use), and early pregnancy complications et al. Second, we acknowledge that the inclusion of pregnant women in south-east China entirely may be a strength in terms of data homogeneity but is a limitation to generalizability. Third, as we were unable to collect data on the timing of SPL incidence, we were unable to perform additional analyses by defining SPL according to the different international criteria to assess the consistency of our findings.^3^

### Conclusion

Our study provides new evidence that abnormal liver function in early pregnancy, as well as elevated GGT and ALP, predisposes pregnant women to an increased incidence of SPL. Such an association may be independent of maternal overweight. Our novel findings identify a new risk factor for SPL that can be modifiable by lifestyle changes and pharmacological interventions. Primary healthcare institutions should be aware of the serious implications of abnormal liver function and offer appropriate preconception counseling to manage this modifiable risk factor in women of reproductive age.

## SUPPLEMENTARY MATERIAL

eFigure 1. The flow chart of this study. MHCIS, Maternal Health Care Information System; SPL, spontaneous pregnancy loss.

eFigure 2. Distribution of maternal liver function biomarkers levels in early pregnancy between SPL and non-SPL. ALP, alkaline phosphatase; ALT, alanine aminotransferase; AST, aspartate aminotransferase; GGT, γ-glutamyl transferase; SPL, spontaneous pregnancy loss.

eFigure 3. Associations of maternal categorical GGT, ALT, AST and ALP with SPL risk. ALP, alkaline phosphatase; ALT, alanine aminotransferase; AST, aspartate aminotransferase; CI, confidence interval; GGT, γ-glutamyl transferase; RR, risk ratio; SD, standard deviation; SPL, spontaneous pregnancy loss.

eTable 1. Clinical reference intervals of the liver enzyme biomarker levels included in this study

eTable 2. Characteristics between pregnant women with or without complete liver enzyme levels data at first antenatal visit

eTable 3. Baseline characteristics of the study population stratified by maternal preconception overweight status

eTable 4. Sensitivity analyses including 709 pregnant women with missing liver enzyme levels after multiple imputation

eTable 5. Subgroup analyses for maternal liver function and SPL risk
