# Supplementary material for: Maternal Abnormal Liver Function in Early Pregnancy and Spontaneous Pregnancy Loss: A Retrospective Cohort Study
Source: J Epidemiol. 2025 May 5;35(5):230–6. doi: 10.2188/jea.JE20240233 (PMC11979345; doi:10.2188/jea.JE20240233)
Supplement: Supplementary file 1 [file je-35-230-s001.pdf]

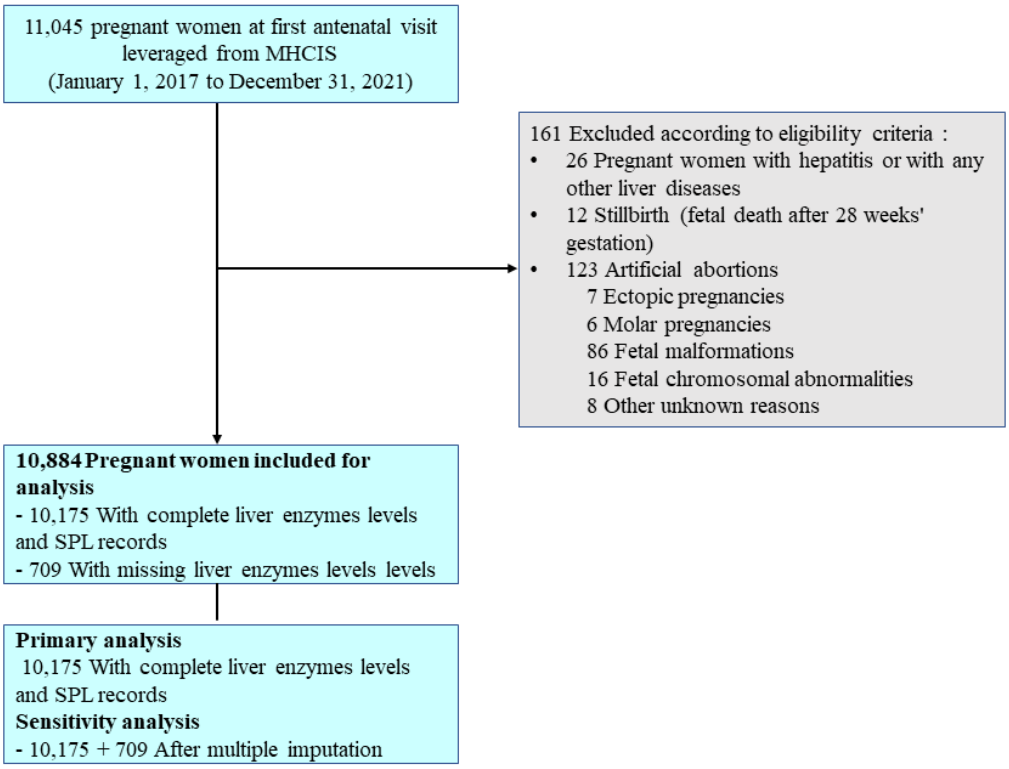

eFigure 1. The flow chart of this study. MHCIS, Maternal Health Care Information System; SPL, spontaneous pregnancy loss.

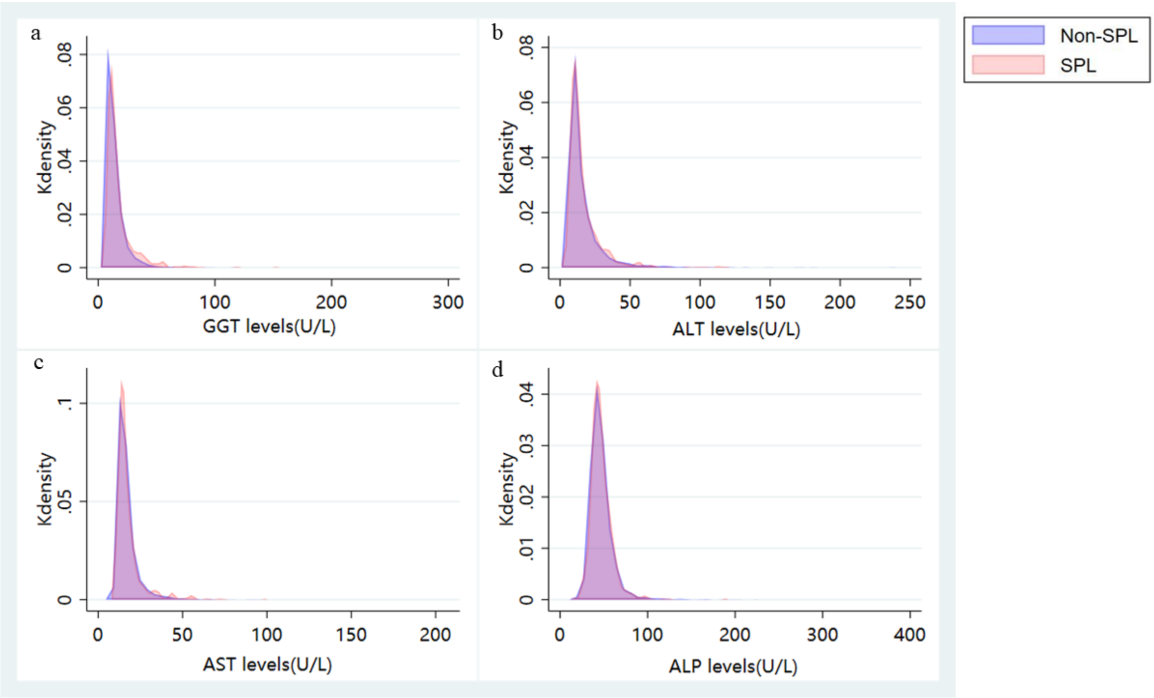

eFigure 2. Distribution of maternal liver function biomarkers levels in early pregnancy between SPL and non-SPL. ALP, alkaline phosphatase; ALT, alanine aminotransferase; AST, aspartate aminotransferase; GGT, γ-glutamyl transferase; SPL, spontaneous pregnancy loss.

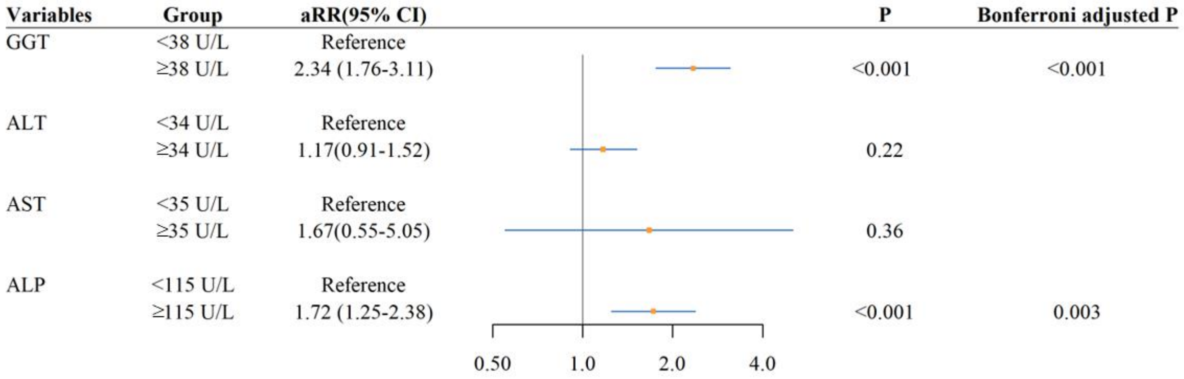

eFigure 3. Associations of maternal categorical GGT, ALT, AST and ALP with SPL risk. ALP, alkaline phosphatase; ALT, alanine aminotransferase; AST, aspartate aminotransferase; CI, confidence interval; GGT, γ-glutamyl transferase; RR, risk ratio; SD, standard deviation; SPL, spontaneous pregnancy loss.

eTable 1. Clinical reference intervals of the liver enzyme biomarker levels included in this study

**Biomarkers**	**Clinical reference intervals**	Abnormal liver function status was defined as having any of the following:
ALT	<34 U/L (Schumann et al. 2002a)	
AST	<35 U/L(Kratz et al. 2004)	GGT ≥38 U/L or ALT ≥34 U/L or
ALP	43–115 U/L (Schumann et al. 2011)	AST ≥35 U/L or ALP ≥115 U/L
GGT	<38 U/L in women (Schumann et al. 2002b)	

ALP, alkaline phosphatase; ALT, alanine aminotransferase; AST, aspartate aminotransferase; GGT, γ-glutamyl transferase.

eTable 2. Characteristics between pregnant women with or without complete liver enzyme levels data at first antenatal visit

Characteristics	pregnant women with complete liver enzyme levels data	pregnant women without complete liver enzyme levels data
N	10,175	709
Age, mean (SD), year	30.1 (4.2)	30.0 (3.9)
BMI, mean (SD), kg/m^2	21.2 (4.0)	21.3 (3.7)
Overweight, n (%)	1,506 (14.8)	93 (13.1)
Missing, n (%)	119 (1.2)	10 (1.4)
Gestational week, mean (SD), week	13.5 (2.9)	14.2 (1.9)
Missing, n (%)	70 (0.7)	5 (0.7)
Parity, n (%)		
0	5,715 (56.2)	446 (62.9)
≥1	4,380 (43.0)	260 (36.7)
Missing, n (%)	80 (0.8)	3 (0.4)
History of abortion, n (%)	2,283 (22.4)	177 (24.9)
Missing, n (%)	150 (1.5)	10 (1.4)
Diabetes before pregnancy, n (%)	12 (0.1)	0
Missing, n (%)	38 (0.4)	2 (0.3)

BMI, body mass index (calculated as weight in kilograms divided by height in meters squared); IQR, inter-quartile range; SD, standard deviation.

Continuous variables are presented as mean (SD) and categorical variables are listed as n (%).

eTable 3. Baseline characteristics of the study population stratified by maternal preconception overweight status

**Characteristics**	**Normal weight (N=8,550)**	**Overweight (N=1,506)**	***P***
Age, mean (SD), year	29.6 (4.1)	30.7 (4.3)	<0.001
Gestational week at first antenatal visit, mean (SD), week	13.5 (2.9)	13.4 (4.3)	0.77
Parity, n (%)			
0	4,849 (57.7)	706 (47.8)	<0.001
≥1	3,553 (42.3)	771 (52.2)	
History of abortion, n (%)	1,892 (22.5)	377 (25.5)	0.020
Missing, n (%)			
GGT, median (IQR), U/L	12.0 (9.0–16.0)	15.0 (11.0–22.0)	<0.001
Elevated levels, n (%)	165 (1.9)	116 (7.7)	<0.001
ALT, median (IQR), U/L	11.00 (9.0–17.0)	14.0 (10.0–22.0)	<0.001
Elevated levels, n (%)	592 (6.9)	165 (11.0)	<0.001
AST, median (IQR), U/L	15.0 (13.0–18.0)	15.0 (13.0–18.0)	0.011
Elevated levels, n (%)	287 (3.4)	44 (2.9)	0.38
ALP, median (IQR), U/L	44.0 (38.0–51.0)	48.0 (41.0–56.0)	<0.001
Elevated levels, n (%)	54 (0.6)	12 (0.8)	0.46
Abnormal liver function, n (%)	762 (8.9)	244 (16.2)	<0.001

ALP, alkaline phosphatase; ALT, alanine aminotransferase; AST, aspartate aminotransferase; GGT, γ-glutamyl transferase; IQR, inter-quartile range; SD, standard deviation.

These characteristics stratified by overweight status were summarized before imputation. Continuous variables are presented as mean (SD) or median (interquartile range), and categorical variables are listed as n (%).

eTable 4. Sensitivity analyses including 709 pregnant women with missing liver enzyme levels after multiple imputation

**Analyses**	**Adjusted RR (95% CI)**	***P* values**	**Bonferroni adjusted *P* values**
Abnormal liver function	1.50 (1.26–1.77)	<0.001	<0.001
GGT (per 1 SD)	1.11 (1.07–1.16)	<0.001	<0.001
ALT (per 1 SD)	1.01 (0.98–1.04)	0.39	0.99
AST (per 1 SD)	1.01 (0.98–1.03)	0.52	0.99
ALP (per 1 SD)	1.13 (1.07–1.19)	<0.001	<0.001

ALP, alkaline phosphatase; ALT, alanine aminotransferase; AST, aspartate aminotransferase; CI, confidence interval; GGT, γ-glutamyl transferase; RR, risk ratio; SD, standard deviation.

eTable 5. Subgroup analyses for maternal liver function and SPL risk

Sub-populations	Adjusted RR (95% CI)	*P* values
**Pregnant women under 35 years (N=9,349)** [a]		
Abnormal liver function	1.58 (1.32–1.90)	<0.001
GGT (per 1 SD)	1.11 (1.06–1.16)	<0.001
ALT (per 1 SD)	1.01 (0.99–1.04)	0.29
AST (per 1 SD)	1.01 (0.99–1.03)	0.38
ALP (per 1 SD)	1.12 (1.06–1.18)	<0.001
**Pregnant women without abortion history (N=7,742)** [b]		
Abnormal liver function	1.38 (1.12–1.80)	0.002
GGT (per 1 SD)	1.10 (1.06–1.16)	<0.001
ALT (per 1 SD)	1.01 (0.98–1.03)	0.72
AST (per 1 SD)	1.01 (0.99–1.03)	0.51
ALP (per 1 SD)	1.12 (1.05–1.18)	<0.001
**LFBs measured in pre-COVID-19 period (N=7,347)** [c]		
Abnormal liver function	1.74 (1.36–2.22)	<0.001
GGT (per 1 SD)	1.13 (1.07–1.19)	<0.001
ALT (per 1 SD)	1.01 (0.99–1.04)	0.22
AST (per 1 SD)	1.01 (0.95–1.07)	0.73
ALP (per 1 SD)	1.14 (1.06–1.21)	<0.001

ALP, alkaline phosphatase; ALT, alanine aminotransferase; AST, aspartate aminotransferase; CI, confidence interval; COVID-19, coronavirus disease 2019; GGT, γ-glutamyl transferase; RR, risk ratio; SD, standard deviation.

These additional association analyses did not correct for multiplicity due to the limited sample size in the subgroup.

[a] Adjusted for gestational week at enrollment, BMI, diabetes before pregnancy, parity and history of abortion;

[b] Adjusted for maternal age, gestational week at enrollment, BMI, diabetes before pregnancy, and parity;

[c] Adjusted for maternal age, gestational week at enrollment, BMI, diabetes before pregnancy, parity and history of abortion.

REFERENCES

Kratz, A., et al., 2004. Case records of the Massachusetts General Hospital. Weekly clinicopathological exercises. Laboratory reference values. N. Engl. J. Med. 351, 1548-63. https://doi.org/10.1056/NEJMcpc049016.

Schumann, G., et al., 2002a. IFCC primary reference procedures for the measurement of catalytic activity concentrations of enzymes at 37 degrees C. International Federation of Clinical Chemistry and Laboratory Medicine. Part 4. Reference procedure for the measurement of catalytic concentration of alanine aminotransferase. Clin. Chem. Lab. Med. 40, 718-24.

Schumann, G., et al., 2002b. IFCC primary reference procedures for the measurement of catalytic activity concentrations of enzymes at 37 degrees C. International Federation of Clinical Chemistry and Laboratory Medicine. Part 6. Reference procedure for the measurement of catalytic concentration of gamma-glutamyl transferase. Clin. Chem. Lab. Med. 40, 734-8.

Schumann, G., et al., 2011. IFCC primary reference procedures for the measurement of catalytic activity concentrations of enzymes at 37 degrees C. Part 9: reference procedure for the measurement of catalytic concentration of alkaline phosphatase International Federation of Clinical Chemistry and Laboratory Medicine (IFCC) Scientific Division, Committee on Reference Systems of Enzymes(C-RSE). Clin. Chem. Lab. Med. 49, 1439-46.
